# Modeling within-Host SARS-CoV-2 Infection Dynamics and Potential Treatments

**DOI:** 10.3390/v13061141

**Published:** 2021-06-14

**Authors:** Mehrshad Sadria, Anita T. Layton

**Affiliations:** 1 Department of Applied Mathematics, University of Waterloo, Waterloo, ON N2L 3G1, Canada; msadria@uwaterloo.ca; 2 Departments of Biology, University of Waterloo, Waterloo, ON N2L 3G1, Canada; 3 Cheriton School of Computer Science, University of Waterloo, Waterloo, ON N2L 3G1, Canada; 4 School of Pharmacy, University of Waterloo, Waterloo, ON N2L 3G1, Canada

**Keywords:** COVID-19, convalescent plasma transfusion, immune response, mathematical modeling, Remdesivir

## Abstract

The goal of this study was to develop a mathematical model to simulate the actions of drugs that target SARS-CoV-2 virus infection. To accomplish that goal, we have developed a mathematical model that describes the control of a SARS-CoV-2 infection by the innate and adaptive immune components. Invasion of the virus triggers the innate immunity, whereby interferon renders some of the target cells resistant to infection, and infected cells are removed by effector cells. The adaptive immune response is represented by plasma cells and virus-specific antibodies. The model is parameterized and then validated against viral load measurements collected in COVID-19 patients. We apply the model to simulate three potential anti-SARS-CoV-2 therapies: (1) Remdesivir, a repurposed drug that has been shown to inhibit the transcription of SARS-CoV-2, (2) an alternative (hypothetical) therapy that inhibits the virus’ entry into host cells, and (3) convalescent plasma transfusion therapy. Simulation results point to the importance of early intervention, i.e., for any of the three therapies to be effective, it must be administered sufficiently early, not more than a day or two after the onset of symptoms. The model can serve as a key component in integrative platforms for rapid in silico testing of potential COVID-19 therapies and vaccines.

## 1. Introduction

The Severe Acute Respiratory Syndrome Coronavirus 2 (SARS-CoV-2) has wreaked havoc all over the world, with a worldwide death toll exceeding 2 million as of January 2021. SARS-CoV-2 has a higher transmission rate than the SARS-CoV, and causes the coronavirus disease 2019 (COVID-19); the new strain appears to be even more infectious. Some COVID-19 patients develop acute respiratory distress syndrome, which has high morbidity and mortality. There is evidence that COVID-19 tends to be more severe in patients with hypertension, diabetes, or advanced age [1], although it is difficult to assess to what extent that preliminary conclusion can be attributable to bias in age, sex, comorbidities, and existing medication. While vaccines have been developed, there is no specific treatment against SARS-CoV-2. Given the rapid spread of COVID-19 and the climbing death toll, identifying effective antiviral agents to combat the disease is urgently needed.

Mathematical modeling has proven instrumental in understanding the global spread of COVID-19, and what measures should be taken to slow that process (e.g., [2,3,4]). Aside from epidemiology studies, mathematical modeling can also be a valuable tool in gaining insights into immune response to infectious diseases [5], by providing a platform for testing hypotheses and revealing biological mechanisms that underly experimental or clinical observations. Mathematical models have been developed for within-host virus dynamics for influenza [6], HIV [7], hepatitis B [8], and hepatitis C [9].

While most of the modeling effort has been directed to epidemiology studies, a number of mathematical models has been developed to describe SARS-CoV-2 in-host dynamics. Ejima et al. model SARS-CoV-2 dissemination among susceptible host cells using a simple 2-ODE system, and apply that phenomenological model to estimate time of infection, and to differentiate imported cases from local secondary cases [10]. Using the same model, Kim et al. simulate potential anti-SARS-CoV-2 therapies [11]. Hernandez-Vargas and Velasco-Hernandez assess the accuracy by which several simple infectious disease models can predict viral dynamics consistent with clinical data [12]. They find that including the immune response substantially improves the fit. Sahoo et al. built a dynamical system model to analyze intra-host dynamics among virally infected cells, and to indent key parameters affecting the diverse clinical phenotypes associated with COVID-19 [13].

The principal goal of this study was to develop a mathematical model of the interactions between SARS-CoV-2 and the immune response, with an ultimate goal of using the model to (1) understand within-host viral dynamics, and (2) assess the effectiveness of potential antiviral therapies and vaccines, including those for variants of concern. As such, the model represents key components of the immune system, including interferon, innate response and adaptive response agents, and predicts viral load and tissue damage over time. The model is carefully calibrated and validated against available clinical data for COVID-19 [14,15]. We then demonstrate the value of the model by simulating three potential COVID-19 therapies (1) Remdesivir, a repurposed drug originally developed for the Ebola virus, (2) an alternative therapy that inhibits SARS-CoV-2′s entry into host cells, and (3) convalescent plasma transfusion therapy.

## 2. Materials and Methods

The present model is based on a dynamical model of human immune response to influenza A viral infection [6]. That model includes the innate immune response, which is represented by interferon-induced resistance to infection of respiratory epithelial cells and by the removal of infected cells by effector cells (associated with cytotoxic T-cells and natural killer cells). The model also includes adaptive immunity, which is represented by SARS-CoV-2-specific antibodies. We have formulated that model to simulate human immune response to uncomplicated SARS-CoV-2 infection.

**Figure 1:**
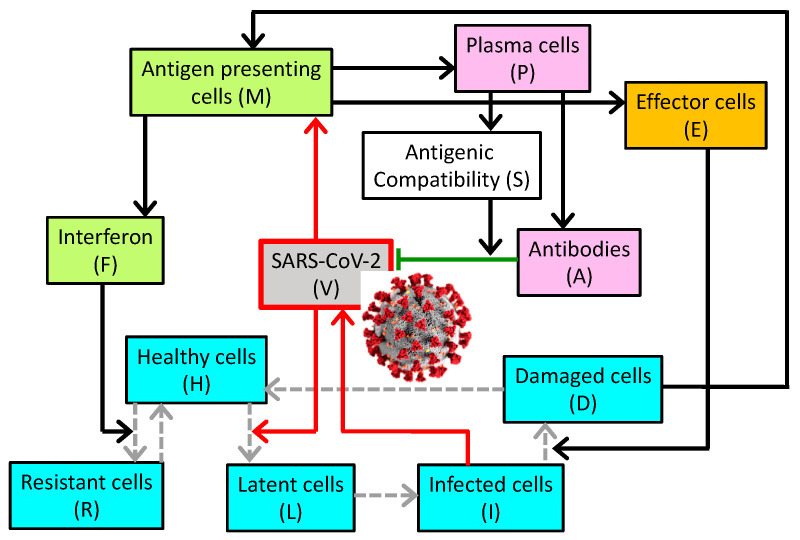
Schematic diagram depicting the interactions among the virus, 5 classes of respiratory epithelial cells (blue boxes), innate immune system (orange), and the adaptive immune system (pink). The conversion from one cell type to another is indicated by gray dashed arrows. Upregulation is indicated by solid black arrows; inhibition by the green line terminated by a bar. Red arrows highlight the direct effects of the virus. The coronavirus image was created at the Centers for Disease Control and Prevention (CDC) and reveals ultrastructural morphology exhibited by coronaviruses.

The model describes the dynamics of SARS-CoV-2 in the general circulation, and the host’s innate and adaptive immune responses. A schematic diagram is shown in Figure 1. The viral load (denoted by V) increases as the viruses reproduce and replicate themselves in infected cells, characterized by a rate constant $\gamma_{V}$. The viral load decreases as the viruses are eliminated by antibodies, denoted by A and characterized by their specificity S and rate constant $\gamma_{VA}$, as the viruses enter healthy cells (denoted by H) and characterized by rate constant $\gamma_{VH}$, as they naturally degrade with rate constant $\alpha_{V}$, and as the viruses are removed by other non-specific mechanisms described by a Michalis–Menten term.

(1) $$\frac{dV}{dt}=\gamma_{V}I-\gamma_{VA}SAV-\gamma_{VH}HV-\alpha_{V}V-\frac{a_{V1}V}{1+a_{V2}V}$$

The population of (susceptible) healthy cells (H) decreases as they are invaded by the virus, at a rate constant of $\gamma_{HV}$. Note that $\gamma_{HV}<\gamma_{VH}$ to represent the possibility of multiple viruses infecting one epithelial cell. Following tissue damage, healing occurs as healthy cells are produced by the proliferation of healthy cells and resistant cells (denoted R); the recovery term is proportional to damage (D) and characterized by rate constant $b_{HD}$. As resistant cells lose their resistance, they become susceptible healthy cells with a rate constant of $a_{R}$. Finally, interferon (F) renders (susceptible) healthy cells resistant with a rate constant of $b_{HF}$ [16].

(2) $$\frac{dH}{dt}=-\gamma_{HV}VH+b_{HD}D\left(H+R\right)+a_{R}R-b_{HF}FH$$

As cells become infected, they first enter an eclipse phrase as “latent” cells (L). With a rate constant of $\gamma_{LI}$, these cells become infected cells capable of facilitating viral replication.

(3) $$\frac{dL}{dt}=\gamma_{HV}VH-\gamma_{LI}L$$

Infected cells (I) may be removed by immune effector cells (E) or undergo natural death, with rate constants $b_{IE}$ and $a_{I}$, respectively.

(4) $$\frac{dI}{dt}=\gamma_{LI}L-b_{IE}EI-a_{I}I$$

Antigen presenting cells (denoted M) are produced when presented with damaged cells or viruses, with proportional constants $b_{MD}$ and $b_{MV}$, respectively. These cells also naturally decay with rate constant $a_{M}$.

(5) $$\frac{dM}{dt}=\left(b_{MD}D+b_{MV}V\right)\left(1-M\right)-a_{M}M$$

Interferon is produced by the antigen presenting cells and infected cells with rate constants $b_{F}$ and $c_{F}$, respectively. Interferon also binds to healthy cells with rate constant $b_{FH}$, and decays with rate constant $a_{F}$.

(6) $$\frac{dF}{dt}=b_{F}M+c_{F}I-b_{FH}HF-a_{F}F$$

As susceptible healthy cells bind to interferon they become resistant. These cells also lose their resistance and become susceptible ($a_{R}R$).

(7) $$\frac{dR}{dt}=b_{HF}FH-a_{R}R$$

The production of effector cells (E) is stimulated by antigen presenting cells, with rate constant $b_{EM}$. Effector cells may be lost in the destruction of infected cells. The last term describes the regulation of the amount of effector cells in the body.

(8) $$\frac{dE}{dt}=b_{EM}ME-b_{EI}IE+a_{E}\left(1-E\right)$$

Similarly, the production of plasma cells (P) is stimulated by antigen presenting cells, and their population is regulated.

(9) $$\frac{dP}{dt}=b_{PM}MP+a_{P}\left(1-P\right)$$

Antibodies (A) are produced by plasma cells with rate constant $b_{A}$. Their population decreases naturally (with death rate constant $a_{A}$) and as they eliminate the viruses (characterized by rate constant $\gamma_{AV}$, which may differ from $\gamma_{VA}$ as multiple antibodies may be required to neutralize a virus.

(10) $$\frac{dA}{dt}=b_{A}P-\gamma_{AV}SAV-a_{A}A$$

S characterizes the specificity of the antibodies to SARS-CoV-2. Its value ranges from 0 (zero compatibility) to 1 (maximal compatibility). During the course of the disease, S increases as plasma cells produce antibodies that are increasingly compatible with viral antigens.

(11) $$\frac{dS}{dt}=rP\left(1-S\right)$$

H, R, L, and I represent the fractions of susceptible healthy cells, resistant cells, latent, and infected cells. The fraction of damaged cells (D) is thus given by

(12) D=1−H−R−L−I

Table 1 contains a list of model parameters and their baseline values, chosen so that viral load peaks approximately 10–12 days after the initial exposure to SARS-CoV-2. We note that the present model is based on a dynamical model of human immune response to influenza A viral infection [6], which has a faster progression with virus tiers peaking 4–5 days after infection. Thus, we initially halved the original kinetic rates, and then selected parameters are further adjusted ($a_{M}$, $a_{V2}$) so that the model describes an uncomplicated SARS-CoV-2 infection. That is, these parameters are fitted so that, given a standard initial viral load, the virus and the infection are cleared within three weeks, as is typical in most COVID-19 patients. The kinetic rates are consistent with [6] and the references therein.

## 3. Results

### 3.1. Simulation of SARS-CoV-2 Infection

We simulate the immune response of a naïve host to SARS-CoV-2 infection. We assume that initially all host cells are healthy and susceptible (i.e., H(0) = 1, L(0) = I(0) = R(0) = D(0) = 0); there are no active antigen presenting cells (M(0) = 0); the initial levels of interferon, effectors, plasma cells, and antibodies are at the homeostatic levels (i.e., F(0) = E(0) = P(0) = A(0) = 1); and the initial specificity is low, such that S(0) = 0.1. Initial SARS-CoV-2 exposure is taken to be V(0) = 0.01.

**Figure 2:**
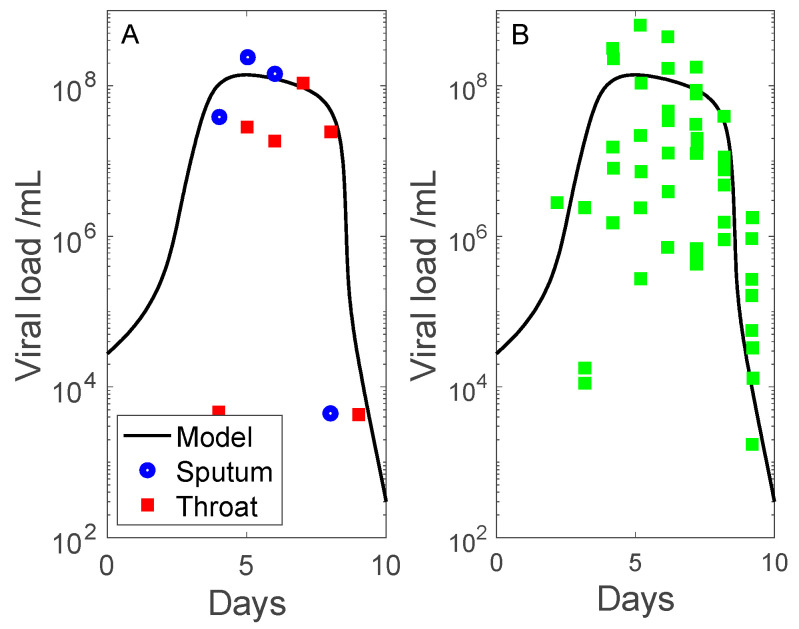
Time-course of viral load, given in days following onset of symptoms, taken to be 7 days following initial viral exposure. Panel (**A**) includes sputum (blue) and throat swab (red) sample data collected by Pan et al. [15]. Panel (**B**) includes sputum viral loads measured by Wolfel et al. [14] in multiple patients (green dots).

**Figure 3:**
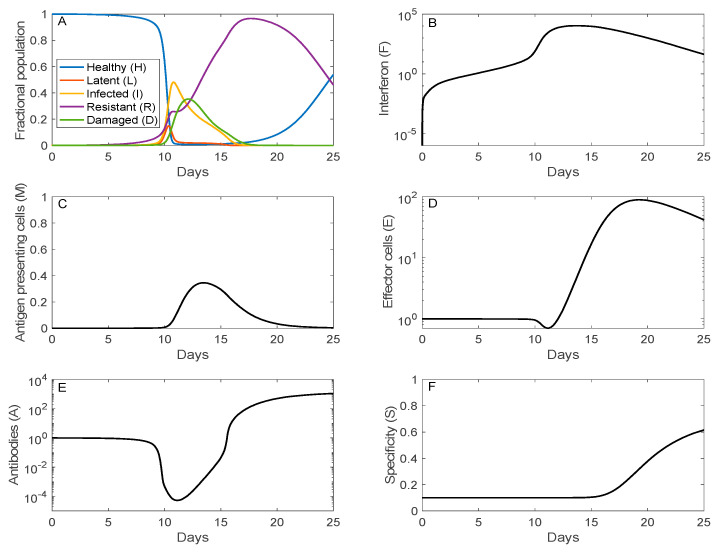
Time-courses of key immune system components as a function of days after initial viral exposure. Panel (**A**), fraction of the five classes of respiratory epithelial cells (healthy, latent, infected, resistant, and damaged). Initially, all cells are susceptible and healthy. A substantial fraction of the cells become infected by day 10. Afterwards, interferon renders most of the cells resistant. As the infection subsides, the cells gradually lose their resistance and become susceptible and healthy. Panels (**B**–**E**), population of interferon, antigen presenting cells, effector cells, and antibodies. Panel (**F**), specificity of antibodies, which increases monotonically over time.

Simulation results are shown in Figure 2 and Figure 3. We compare the predicted viral load with measured data from two clinical studies. The first dataset contains throat swab and sputum sample data collected by Pan et al. [15] (obtained in patient 1, adjusted so that undetectable viral load corresponds to 10^2^–10^3^ copies per mL). The second dataset contains sputum viral loads measured by Wolfel et al. [14] (sputum samples in multiple patients). Data are shown in days after onset of symptoms, with symptoms assumed to begin on day 7. The measured viral load data exhibit a substantial range, even with outliers removed. The discrepancies may be attributed to differential viral exposure, and inaccuracies inherent in throat swabs and sputum specimens [17]. Nonetheless, the model predicts viral load dynamics that share substantial similarities with clinical data: (i) viral load peaks 7 days after onset of symptoms, and (ii) the infection is cleared (viral load approaches zero) 10 days after onset of symptoms. 

The time trajectories of the fraction of host cells that are healthy, infected (latent or infectious), resistant, or damaged are shown in Figure 3A. Just over half of the host cells become infected (I + L) around day 11 (following initial viral exposure, not onset of symptoms). The population of damaged cells reaches its peak at 35% on day 12. Afterward, the recovery phrase begins, with increasingly more cells becoming resistant to infection. The resistant cells eventually lose their resistance and become susceptible healthy cells. 

Maximal interferon response (10^4^-fold above its homeostatic level) coincides with the peak of the viral load around day 12, rendering most of the cells resistant to infection. The elevated viral load and accumulation of damaged cells activate antigen presenting cells after day 10 (panel D), which in turn stimulate the production of effector cells and plasma cells. Production of both effector and plasma cells is negligible until day 11, peaking at day 20 (see panel D for effector cell dynamics). The increase in antibodies lags that of plasma cells, consistent with the experimental data in Ref. [18], climbing to 10^3^-folds above its homeostatic level on day 25. The antibodies are responsible for the clearance of the viruses. Furthermore, antigenic compatibility (panel F) increases monotonically, reaching 62% compatibility on day 25.

### 3.2. Sensitivity Analysis

Many of the model parameters are not well characterized and their baseline values have substantial uncertainties. To gain insights into how variations in parameter values affect model predictions, we perform a sensitivity analysis. Model parameters (Table 1) are varied individually by ±20%. Model equations are solved for each parameter set to determine key outputs that characterize the severity of the disease: maximum viral load (V) and the corresponding time, which is assumed to correlate with disease onset, as well as maximum cell damage (D).

Our results indicate that disease severity is particularly sensitive to variations in the following parameters. The rate constants for viral production $\gamma_{V}$ and for viral infection of cells $\gamma_{HV}$ are positively correlated with disease severity. The larger these rates, the higher the peak viral load (increased by ~25% with +20% increase in rate constants), the earlier the onset of the disease (decrease by ~4 days), the more extensive the cell damage (increase by ~15%), and vice versa. Other parameters such as the infected cell death rate $a_{I}$ exert opposite effects. Increasing $a_{I}$ by 20% reduces peak viral load by 12%, although the impact on disease onset and cell damage is minimal. Disease severity is relatively insensitive to variations in other parameters such as the rate of plasma cell production ($b_{PM}$) or the rate of loss of antigen presenting cells ($a_{M}$). The substantial sensitivity of model predictions to variations in some parameters may explain the large disparity in onset, duration, and severity of SARS-CoV-2 infection.

### 3.3. Simulation of Remdesivir

Remdesivir (GS-5734) is a nucleotide analog prodrug, originally developed for Ebola [19], that has been shown in animal models and in vitro studies to be effective against Middle East Respiratory Syndrome (MERS)-CoV, SARS-CoV, and SARS-CoV-2 infection [20,21,22,23]. Here, we assess the effectiveness of Remdesivir against human SARS-CoV-2 infection. To that end, we first estimate the effect of Remdesivir on viral dynamics by simulating the experiment by Williamson et al. in *rhesus macaques* [22]. Remdesivir inhibits viral transcription rate. We simulate that effect by reducing the viral replication by infected cells ($\gamma_{V}$,). To determine x, we simulate the experimental protocols in Ref. [22], in which Williamson et al. administered Remdesivir to rhesus macaques 12 h after their exposure to SARS-CoV-2. To simulate the more acute nature of SARS-CoV-2 infection in rhesus macaques [22], we assume an larger initial viral load V(0) = 1, which corresponds to an initial concentration of aerosol delivered virus particles that the host receives is about 10^10^ particles per mL on day 0.

**Figure 4:**
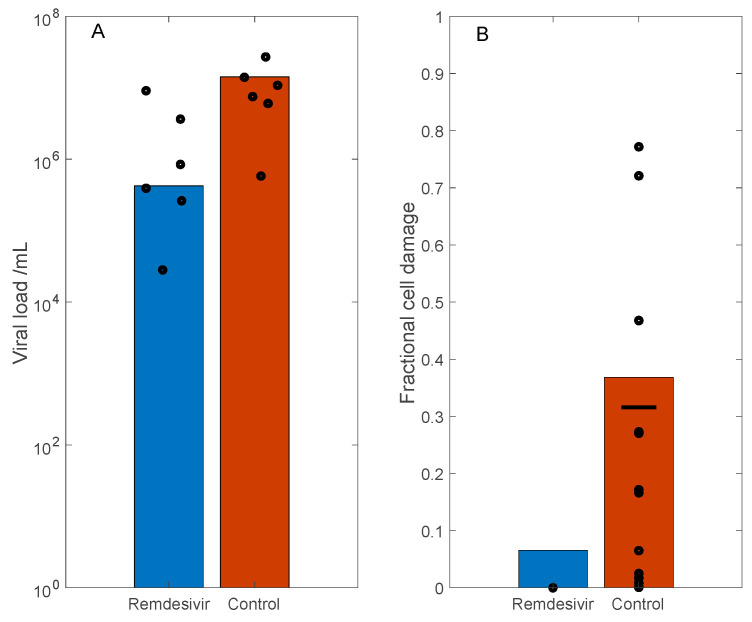
Simulation of SARS-CoV-2 dynamics in rhesus macaques, and the effect of Remdesivir, which inhibits viral transcription. Remdesivir was administered 12 h following initial viral exposure. Predicted viral load (Panel (**A**)) and fractional cell damage (Panel (**B**)), obtained for control (untreated) and Remdesivir-treated case, 3 days after initial exposure. Circles correspond to data from Ref. [22]. Horizonal bar in panel (**B**) denotes mean values of control data.

In the rhesus macaque experiments, the measured viral loads of the control group are approximately two orders of magnitude larger than the treated groups (Figure 2 and Figure 4 in Ref. [22]). Based on these data, we simulate the antiviral effect of Remdesivir by reducing the viral replication by infected cells ($\gamma_{V}$) by 90%, which corresponds to reported efficacy of ~5 μM Remdesivir in the in vitro study by Wang et al. [23] (Figure 1, panel a). With these parameters, on day 3, the model predicts a two orders of magnitude difference in viral load between the control and treated groups. These results are shown in Figure 4A, together with the viral load measurements in rhesus macaque bronchoalveolar lavage fluid, obtained 3 days post inoculation (data extracted from Figure 2A in Ref. [22], adjusted for human size). Moreover, the model predicts 37% and 7% cell damage in control and the treated case, respectively. These results are shown in Figure 4B, and compared with the fractional area affected by gross lesions on the dorsal surface of the left lung lobe of the rhesus macaques (Figure 5A in Ref. [22]). These model predictions are largely consistent with experimental data.

**Figure 5:**
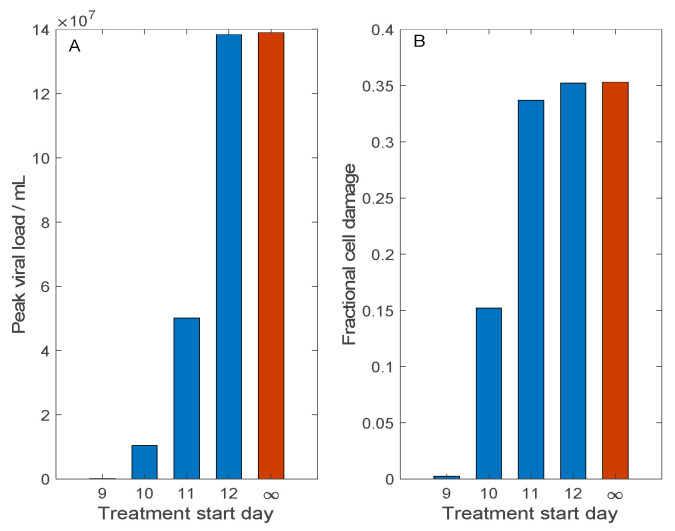
Effect of Remdesivir treatment on SARS-CoV-2 dynamics, and how that effect changes with treatment delay. Treatment may begin 9, 10, 11, or 12 days after initial viral exposure. Predicted maximum viral load (Panel (**A**)) and fractional cell damage (Panel (**B**)) are shown. Blue bar, with Remdesivir treatment; red bars, control (no treatment). Remdesivir offers significant protection only if administered no more than 10 days following exposure, or almost immediately after symptom onset.

In the above experiments [22], Remdesivir was administrated 12 h following viral exposure. However, in practice, an infected person typically seeks help after the onset of symptoms, which may take 3–14 days after exposure to SARS-CoV-2. To assess the effectiveness of this treatment when it is administered with a significant delay, we conduct simulations in which Remdesivir is administrated 9–12 days after initial viral exposure. Here, we simulate a typical human exposure to SARS-CoV-2 and not the more acute infection in rhesus macaques; thus, the baseline initial viral load is used, with V(0) = 0.01. The predicted peak viral load and fraction of damaged cells on day 12 (after viral exposure) are shown in Figure 5. Consider first the case when Remdesivir is administered 9 days after exposure. This time frame corresponds to a couple of days after onset of symptoms, and results in viral clearance (Figure 5A) and minimal tissue damage (Figure 5B). However, with another day of delay, there is 15% tissue damage. Any additional delay (>10 days after initial exposure) would render Remdesivir largely ineffective. The ineffectiveness of Remdesivir in these cases can be explained by its mechanism of action: Remdesivir inhibits viral transcription rate. Given a sufficient delay of drug administration, the viral load has reached a sufficiently high level; thus, any inhibition of transcription rate by Remdesivir has minimal effect.

### 3.4. Host Cell Entry Inhibition

**Figure 6:**
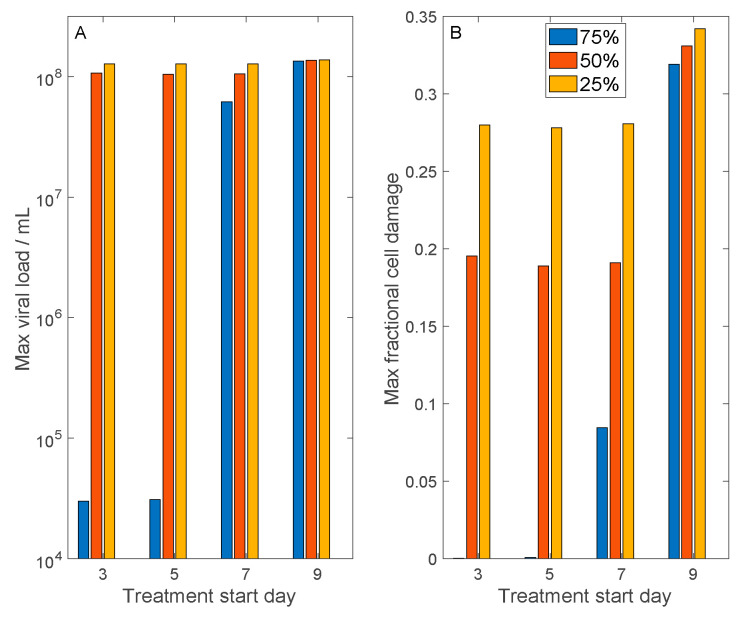
Model simulations to assess the results of antiviral therapies that inhibit cell entry by SARS-CoV-2. Considered are therapies that inhibit cell entry by 75%, 50%, and 25%. Treatment may begin 3, 5, 7, or 9 days after initial exposure to SARS-CoV-2. Panel (**A**), predicted maximum viral load. Panel (**B**), predicted maximum fractional cell damage. For the 75%-effective treatment, if applied within a week after exposure (or almost immediately after onset of symptoms), tissue damage may be limited to <10%. For the 50%-effective treatment, a similar timeline would limit tissue damage to ~20%. The 25%-effective treatment offers little protection.

An alternative antiviral therapy suppresses viral development by inhibiting their entry into host cells. Both SARS-CoV and SARS-CoV-2 gain entry into host cells via the binding of their spike proteins with a membrane receptor, angiotensin converting enzyme 2 (ACE2). We simulate the effect of this class of antiviral therapies by inhibiting SARS-CoV-2 cell entry by differing degrees: by 75%, 50%, and 25%. In the model, this is done by reducing $\gamma_{VH}$ and $\gamma_{HV}$ (Equations (1) and (2)). We also consider the initiation of the therapy with a range of delays: 3, 5, 7, and 9 days following initial viral exposure. For each case, we computed maximum viral load and maximum fractional cell damage. The results are shown in Figure 6. 

For the more effective drug that inhibits viral cell entry by 75%, if administered sufficiently early (within 5 days after exposure), the host suffers essentially no cell damage. Even if the drug is administered 7 days after exposure, maximum cell damage is limited to <9%. However, if the drug is administered more than 9 days after exposure, maximum cell damage is similar to the untreated case (~35%). For the medium effective drug that inhibits viral cell entry by 50%, if it is administered a week or less following viral exposure, then cell damage can be limited to <20%, even though the maximum viral load is not significantly reduced. However, a longer delay would render the treatment ineffectively. A less effective drug that inhibits viral cell entry by 25% has only limited protective effect on host cells.

### 3.5. Convalescent Plasma Transfusion Therapy

Immunotherapy with neutralizing antibodies present in convalescent plasma has been used to treat patients with severe COVID-19. Recovery was reported in two preliminary studies, one by Shen et al. involves 5 patients at the Shenzhen Third People’s Hospital [24] and the other by Duan et al. involves 10 patients from three participating Chinese hospitals [25]. We conduct simulations to understand the determinants for success for convalescent plasma therapy. 

To model convalescent plasma transfusion, we add a new variable A* to represent SARS-CoV-2 specific antibodies in donor plasma. The rate of change of A* is given by

(13) $$\frac{dA^{*}}{dt}=b_{A}^{*}-\gamma_{AV}A^{*}V-a_{A}A^{*}$$

where $b_{A}^{*}$ is a source term that represents plasma transfusion. $b_{A}^{*}$ is set to MA*/TA* during the transfusion period T_A__*_, where M_A__*_ denotes the total amount of SARS-CoV-2 specific antibodies present in donor plasma; outside of this period, $b_{A}^{*}$ is 0. A* is assumed to have maximum specificity (i.e., S implicitly equals 1). The action of A* is taken into account in the viral evolution equation

(14) $$\frac{dV}{dt}=\gamma_{V}I-\gamma_{VA}\left(SA+A^{*}\right)V-\gamma_{VH}HV-\alpha_{V}V-\frac{a_{V1}V}{1+a_{V2}V}$$

Donor plasma antibody titer varies widely, by as much as an order of magnitude (see table 3 in Ref. [24]). Thus, we simulate a range of initial donor A*: 10, 25, 50, and 100 times the homeostatic antibody level. We further consider treatment delay, starting the transfusion 8 to 11 days after initial viral exposure, which corresponds in this model to approximately 1 to 4 days after symptom onset.

**Figure 7:**
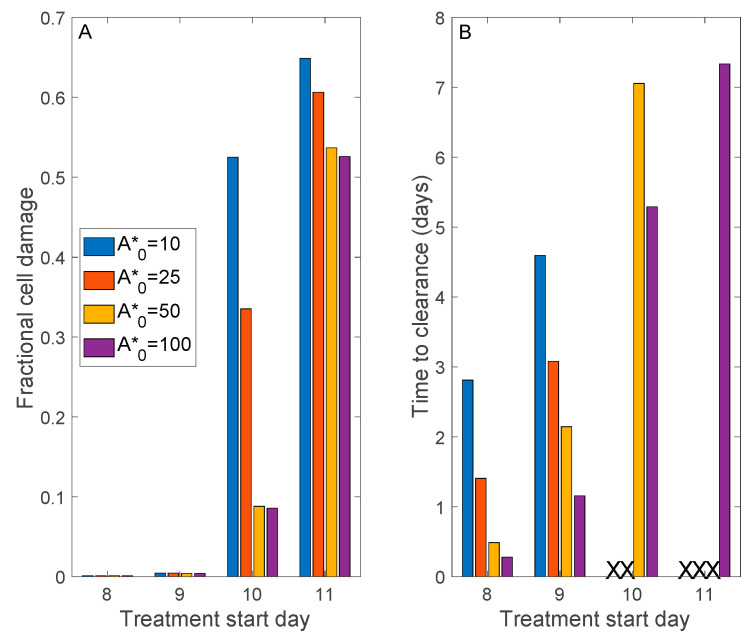
Model simulations to assess the effectiveness of convalescent plasma therapy. A range of donor plasma antibody titers (A*_0_) and treatment delay (after initial viral exposure) are considered. Panel (**A**), maximum fractional cell damage; Panel (**B**), number of days after initiation of therapy to viral clearance. ‘X’ denotes no clearance. If treatment begins sufficiently early, all A*_0_ values considered yield viral clearance and essentially no tissue damage was found. Otherwise, a sufficiently high donor plasma antibody titer would be required to clear the infection. Severe tissue damage may not be avoided if treatment begins more than 10 days after viral exposure.

Figure 7 shows predicted peak tissue damage and time to viral clearance (defined by *V* < 0.01). If the patient is treated sufficiently early, no more than 9 days following viral exposure, all donor plasma antibody levels result in viral clearance and essentially no tissue damage. However, further delays would require a sufficiently high donor plasma antibody titer (initial $A^{*}\geq \;$50) to limit cell damage to <10%; otherwise, therapy fails to attain viral clearance (Figure 7B). If therapy begins on the 11th day, viral clearance is achieved only for the highest donor plasma antibody titer. However, severe tissue damage is not avoided (>50% damage), even with the highest donor plasma antibody titer (Figure 7A). The model predicts that if the therapy is to result in viral clearance, it would happen within a week following therapy (Figure 7B), a result that is in general agreement with preliminary clinical findings [24,25].

## 4. Discussion

The availability of SARS-CoV-2 vaccines is a relief for many. Nevertheless, the virus has undergone and will continue to undergo mutation. Indeed, “third waves” caused by variants of concern have emerged in many countries. Furthermore, SARS-CoV-2 is almost surely not the last novel coronavirus we must battle. Thus, a modeling platform that can facilitate in silico drug testing will be of tremendous value. To advance towards that goal, we have developed a detailed mathematical model of within-host dynamics of SARS-CoV-2. The model represents target cells, divided into five classes (healthy, latent, infected, resistant, and damaged), interferon, innate immune components, and adaptive immune components. The model is based on a published model of influenza A [6], with model parameters refitted to produce a viral load time-course consistent with COVID-19 patient data [14,15]. The 6-h eclipse period of a COVID-19 infection has also been added [26]. The present model predicts the invasion of target cells by SARS-CoV-2, the activation of interferon and its effects, the attack of SARS-CoV-2 by the host’s immune response, the production of tissue damage, and (with some parameters) eventual recovery.

The present model represents a substantially greater degree of details than the published COVID-19 models [10,11,12,13]. We believe that a mathematical model should have as many components as needed for its intended use, but not much more. Our goal is to develop a model of SARS-CoV-2 infection that can be used to simulate the effects of potential therapies and new vaccines, including those for variants of concern. Given that vaccines and likely some of the therapies will target the immune system, we base our model on an infectious disease model that explicitly and separately represents the innate and adaptive immune response [6]. There are mathematical models that represent the immune system in even greater details (e.g., [27]). In addition, the present model does not predict the COVID-19-induced cytokine storm or other complications. Certainly, the model can be extended as needed in the future.

Using the model, we conduct in silico testing of three potential anti-SARS-CoV-2 therapies. We assess the effectiveness of Remdesivir, which was originally developed by Gilead Sciences as a treatment for Ebola virus disease and Marburg virus infection [19]. Remdesivir attempts to halt the spread of the virus by inhibiting its transcription. Our simulation results indicate that for Remdesivir to be effective, it must be administered sufficiently early, not more than a day or two after the onset of symptoms (Figure 5). 

A similar conclusion is drawn when we simulate an alternative (hypothetical) anti-SARS-CoV-2 therapy that inhibits its entry into host cells. SARS-CoV-2, as well as its predecessor SARS-CoV, invade host cells by binding with the membrane receptor ACE2. ACE2 is a key component of the renin-angiotensin system (RAS) and is found on the cells of a number of tissues, including the type 2 alveolar epithelial cells in the lungs [28]. Thus, drugs that reduce ACE2 activity may slow the invasion of host cells by SARS-CoV-2. However, given the anti-inflammatory benefits of ACE2, its inhibition may have significant side effects. In silico testing of viral entry inhibitors again points to the importance of early intervention (Figure 6).

We also consider convalescent plasma therapy, a classic adaptive immunotherapy that has been proven successful in the treatment of SARS, MERS, and 2009 H1N1 pandemic with satisfactory efficacy and safety [29,30,31,32]. In contrast, the convalescent plasma therapy was unable to significantly improve the survival in the Ebola virus disease. That failure may be attributed to the absence of data of neutralizing antibody titration for stratified analysis [33]. Given the similarities between SARS, MERS, and COVID-19, in terms of virological and clinical characteristics, the convalescent plasma therapy might be a promising treatment option for COVID-19 [34]. Indeed, preliminary studies conducted in Chinese hospitals have reported encouraging results [24,25]. Our model simulations indicate that early intervention with sufficiently high donor plasma antibody titers is key to success (Figure 7).

A common message among all sets of three treatment simulations is that for these treatments to be effective, they must be applied very early. This may be particularly true for therapeutic strategies intended to limit the access and intracellular replication of the virus, since the onset of symptoms typically appear following the intracellular multiplication of the virus. This fact likely severely limits the potential clinical effectiveness of therapies targeting this phase of viral pathogenesis, and may explain the subpar efficacy of Remdesivir as a COVID-19 cure.

One of our motivations for developing the present model is to understand risk factors that predispose a subpopulation to more severe sequela for COVID-19. Current data indicate that fatality rates are higher for patients with hypertension (6%), diabetes (7.3%), cardiovascular disease (10.5%), and age >70 (10.2%) [1], although it is difficult to assess to what extent that preliminary conclusion can be attributable to bias in age, sex, comorbidities, and existing medication. Nonetheless, there has been concern that some anti-hypertensive treatments, specifically RAS inhibitors, may increase the risk of SARS-CoV-2 infection and lead to more severe COVID-19 owing to the aforementioned RAS-mediated cell entry mechanism of the virus [35]. Given the success of these RAS inhibitors (the angiotensin converting enzyme (ACE) inhibitors and angiotensin II receptor blockers (ARBs)) in treating cardiovascular diseases, the decision to discontinue their use, or not, should not be made lightly. The present model can be expanded to represent the binding of SARS-CoV-2 to the appropriate membrane receptors to gain cell entry. The resulting model can be used to assess the extent to which ACE inhibitors and ARBs promote the internalization of SARS-CoV-2 and predispose the host to more severe COVID-19.

The clinical relevance of our analysis and conclusions may be limited by the simplifications present in the model. For instance, to answer the above question regarding the interactions between RAS inhibitors and SARS-CoV-2, one may combine the present model with models that describe the renin-angiotensin system [36], ACE2 dynamics [37], and cardiovascular function [38,39]. Another limitation is that the nature of SARS-CoV-2 and our immune response remains poorly described; as such, many of the model parameters are not well characterized and are derived from influenza. In particular, the efficiency of the existing antibodies to neutralize the virus is represented by the variable *S,* which describes the probability of a match between the existing antibodies and the antigenic structure of SARS-CoV-2. Even though *S* is a major determinant in the severity of the infection, its representation in the present is likely overly simplistic (Equation (11)), with the learning rate *r* inadequately characterized. A more sophisticated model of antigen distance would yield more accurate model predictions. A more detailed model that explicitly represents different types of cytokines and lymphocytes can yield predictions that can be compared with observed trajectories of cytokine profiles and lymphocyte responses in COVID-19 patients [40,41]. In addition to the type 2 alveolar epithelial cells in the lungs, several cells expressed ACE2, including the proximal tubule and glomerulus in the kidney [42,43], brain [44], and gut [45]. For simplicity, the present model is developed for a generic cell that expresses ACE2 and does not consider the influence of these different target cells in different tissues. Including cell specificity would provide useful information to resolve a prognostic symptom, which is often a major challenge in COVID-19. In addition, T cells are known to play key roles not only in developing immunity to COVID-19, but severe sequela as well. Indeed, hyperactivation of pro-inflammatory cytokines produced by cytotoxic T cells is known to contribute to the severity of COVID-19. However, T cell responses in COVID-19 remain to be determined, with evidence existing that supports suboptimal or excessive responses [46]. Once T cell dysregulation in COVID-19 and the underlying molecular mechanisms are better characterized, the present model would be enhanced by incorporating the role of excessive pro-inflammatory signals in severe COVID-19 sequela. Finally, a more realistic representation of tissue damage (D) would allow the model to be used to simulate the effects of anti-inflammatory agents such as steroids. 

Despite its limitations, the SARA-CoV-2 infection model presented in this study provides a basis for the development of a much-needed platform for in silico testing of potential therapies and future vaccines for COVID-19. The model can also be used to predict viral shedding, which can be related to viral load. This extension would characterize the contagious period and allow the model to predict the spread of the disease at a population level. A more refined model that incorporates the patient’s sex [47,48], age [49], and concurrent therapies (e.g., for diabetes [50,51,52]) would be a valuable diagnostic tool.

## Figures and Tables

**Table 1:** Model parameters.

Symbol	Description	Value
$\gamma_{V}$	Viral production by infected cells	255
$\gamma_{VA}$	Elimination of virus by antibodies	309.6
$\gamma_{VH}$	Virus entering healthy cells	0.51
$\alpha_{V}$	Virus degradation	0.85
$a_{V1}$	Maximal rate of virus removal	50
$a_{V2}$	Michaelis–Menten constant in virus removal	23,000
$\gamma_{LI}$	Latent cells becoming fully infected	6
$b_{HD}$	Regeneration of epithelial cells	2
$a_{R}$	Loss of viral resistance	0.5
$\gamma_{HV}$	Rate of infection of cells by virus	0.17
$b_{HF}$	Susceptible cells becoming viral resistant	0.005
$b_{IE}$	Infected cells damaged by effector cells	0.033
$a_{I}$	Infected cells death rate	0.775
$b_{MD}$	Stimulation of antigen presenting cells by damaged cells	0.5
$b_{MV}$	Stimulation of antigen presenting cells by virus	0.00185
$a_{M}$	Antigen presenting cell natural death	0.75
$b_{F}$	Interferon production rate per antigen presenting cell	125,000
$c_{F}$	Interferon production rate per infected cell	1000
$b_{FH}$	Interferon binding to susceptible healthy cells	8.5
$a_{F}$	Interferon natural decay	4
$b_{EM}$	Stimulation of effector cells	4.15
$b_{EI}$	Death of effector cells by infector cells	1.36
$a_{E}$	Effector cell natural death	0.2
$b_{PM}$	Plasma cell production	5.75
$a_{P}$	Plasma cell natural death	0.2
$b_{A}$	Antibody production rate per plasma cell	0.0225
$\gamma_{AV}$	Antibodies binding to viruses	73.1
$a_{A}$	Antibody natural death	0.0215
*r*	Change in antibody specificity	0.000015

## Data Availability

MATLAB programs used in the model simulations can be accessed at https://github.com/MehrshadSD/COVID19-immune-system.git (accessed on 18 April 2021).
